# Advances in green chemistry and engineering

**DOI:** 10.1038/s41598-024-53594-z

**Published:** 2024-03-07

**Authors:** Jechan Lee, Assunta Marrocchi

**Affiliations:** 1 Department of Global Smart City & School of Civil, Architectural Engineering, and Landscape Architecture, Sungkyunkwan University, Suwon, 16419 South Korea; 2 Department of Chemistry, Biology and Biotechnology, University of Perugia, 06123 Perugia, Italy

**Keywords:** Environmental impact, Climate-change mitigation

## Abstract

Green chemistry and engineering seek for maximizing efficiency and minimizing negative impacts on the environment and human health in chemical production processes. Driven by advances in the principles of environment protection and sustainability, these fields are expected to greatly contribute to achieving sustainable development goals. To this end, many studies have been conducted to develop new approaches within green chemistry and engineering. The Advances in Green Chemistry and Engineering Collection at *Scientific Reports* aims at gathering the latest research on developing and implementing the principles of green chemistry and engineering.

## Introduction

In the research areas of green chemistry/engineering, green synthesis of chemicals and solvents, waste recycling, and biodegradable polymer synthesis are emerging trends. Developing less hazardous synthesis protocols of chemicals and solvents is necessary to move the industrial landscape in various sectors (organic synthesis, pharmaceuticals, pollutant extraction, etc.) forward to a long-term sustainable reality. Although earlier studies on green synthesis of chemicals/solvents tended to focus on small molecules, medium-sized molecules have recently garnered more attention. The current landscape of waste recycling is to recover value-added fractions (e.g., monomers and functional materials) from complex waste substances characterized by intricate compositions (e.g., composite and unsorted plastics). Synthesized biodegradable polymers have continuously gained attention due to the difficulty in procuring reproducibility when using natural polymeric materials^1^. Many recent studies aim to improve the scalability and stability of biodegradable polymers to meet criteria for commercialization.

### Article highlights in this collection

Highly-stable low-toxic organogermanium compounds have recently been used as reagents in the synthesis of complex organic molecules^2,3^. Stachowiak‑Dłużyńska et al. reported an efficient protocol for the reactions between alkynylgermanes and silanols or terminal acetylenes using a commercially-available potassium bis(trimethylsilyl)amide (KHMDS) as the catalyst^4^. The KHMDS catalyst allowed a dealkynative coupling to synthesize a variety of organogermanes such as alkynylgermanes and germasiloxanes. The KHMDS-based process offered desirable features such as high chemoselectivity, simple operation, benign reaction conditions, and low cost of the reagents and catalyst, thereby potentially enabling green synthesis of value-added organogermanium compounds.

Natural deep eutectic solvent (NADES), made from non-toxic components derived from natural compounds (e.g., menthol, thymol, organic acids, and salts)^5^, is considered an environmentally friendly solvent. Hunter et al. proposed a method of extracting micro‑ and nano‑plastic particles from water using hydrophobic NADES^6^. Three hydrophobic NADESs were made by combining decanoic acid, menthol, or thymol with different molar ratios. They were used to extract micro‑ and nano‑plastic particles of polystyrene, polyethylene terephthalate, and polylactic acid from fresh water and salt water. The extraction efficiencies ranged between 50 and 93% within 0.2–1.3 h. The extraction efficiency was highly associated with the type and size of plastic particles.

Among various coating methods, thin phosphate-based coating combines crucial material protection potential and environmental benefits by repelling water, decomposing organic and inorganic compounds, and providing anticorrosive properties^7^. Zemajtis et al. investigated the effect of a hydrophobic agent on the transformation from superhydrophilicity to superhydrophobicity of TiO_2_-doped zinc phosphate coating systems using high-resolution neutron imaging (HR-NI), confocal laser scanning microscope, scanning electron microscope, and X-ray diffraction techniques^8^. The HR-NI showed that water imbibition is observed for the superhydrophilic coating, while water ingress into porous ceramic substrate is prevented by the superhydrophobic coating. The characteristics of the TiO_2_-doped zinc phosphate coating were highly associated with chemical bonding, surface roughness, and photocatalytic reactivity. It was also demonstrated that a two-layer superhydrophobic system can create effective water barriers on the surface with the contact angle of 153° that is still effective for damaged surface.

Due to rapidly growing demand for wind energy, the generation of wind turbine blade waste have increased. Therefore, decommissioned wind turbine blades need to be carefully recycled^9^. Muzyka et al. proposed a novel concept of end-of-life wind turbine blade recycling process^10^. The process is to dissolve thermoset resin fraction (containing ester groups) of wind turbine blade waste and carbon fiber composites via transesterification at < 200 °C in the presence of 1,5,7-triazabicyclo[4.4.0]dec-5-ene, ethylene glycol, and 1-methyl-2-pyrrolidinone (1:1:1 molar ratio), resulting in up to 100% resin degradation yield. Fiber materials could be readily separated from the product stream. The resin degradation yield may depend on waste compositions.

Finding alternatives to non-biodegradable plastics has raised concerns worldwide as plastic waste harms the environment. Microalgae are considered as a renewable source for bioplastic production^11^. Chalermthai et al. conducted techno‑economic assessment (TEA) of co‑production of food packaging bioplastic and food supplements from edible microalgae, *Arthrospira* spp. (Spirulina)^12^. In their study, the production scale was assumed to meet 1% of the local plastic demand in Thailand (≈1200 MT per year) and 1% of the global Spirulina demand as food supplement (≈1000 MT per year). The TEA results indicated that the co-production of the Spirulina-based bioplastic and food supplement powder is an attractive approach with the annual revenue of up to USD 55.6 million, a payback time of ≈2.6 y, and a return on investment of ≈38.5% despite high capital and operating costs. In the large-scale process, the selling price of bioplastic and the split ratio of biomass used for bioplastic and food supplement were found to be the most sensitive parameters affecting its profitability.

This Collection provides a group of articles offering new insights into the approaches to synthesize less hazardous functional chemicals and solvents for different applications (e.g., organic synthesis, microplastic extraction, and coatings), suggesting new routes to develop composite waste recycling method, and evaluating the techno-economic potential of algal biorefinery for producing both bioplastic and food supplement.
